# Lung ultrasound to guide the administration of exogenous pulmonary surfactant in respiratory distress syndrome of newborn infants: A retrospective investigation study

**DOI:** 10.3389/fped.2022.952315

**Published:** 2022-10-12

**Authors:** Jing Liu, Wei Fu, Shen-Juan Qin

**Affiliations:** ^1^ Department of Neonatology and NICU, Beijing Chao-Yang Hospital, Capital Medical University, Beijing, China; ^2^ Department of Neonatology and NICU, Beijing Chaoyang District Maternal and Child Healthcare Hospital, Beijing, China

**Keywords:** lung ultrasound (LUS), pulmonary surfactant (PS), respiratory distress syndrome (RDS), neonate, lung disease, European RDS management guideline, chest x-ray (CXR)

## Abstract

**Background:**

Respiratory distress syndrome (RDS) is a common disease that seriously endangers the life and safety of newborns, especially premature infants. Exogenous pulmonary surfactant (PS) is the specific agent for the treatment of neonatal RDS. Lung ultrasound (LUS) has been successfully used in the diagnosis of RDS, but its value in guiding the application of PS is still unclear. This paper explored whether the application of PS under LUS monitoring has some advantages, including (1) decreasing the misdiagnosis rate of RDS and decreasing probability of using PS, and (2) reducing the dose of PS without reducing the therapeutic effect.

**Methods:**

This study included two parts. Part 1: To decide whether the LUS is good to differentiate RDS from other lung diseases in the premature infants. All patients who were diagnosed with RDS and required PS treatment based on conventional criteria were routinely examined by LUS. Then, according to LUS findings, we decided whether they needed to receive PS treatment. Part 2: To see the dose reduction of surfactant is applicable. In RDS patients diagnosed based on LUS presentation and treated with Curosurf (Chiesi Pharmaceutical, Parma, Italy), the dose of Curosurf was compared with that recommended by the European RDS management guidelines.

**Results:**

(1) Since March 2017, 385 newborn infants admitted to our neonatal intensive care unit met the traditional diagnostic criteria of RDS. Of these, only 269 cases were diagnosed with RDS and needed PS treatment according to LUS manifestations. The other 116 infants who did not meet the criteria for ultrasound diagnosis of RDS did not receive PS supplementation but obtained good outcomes, that is LUS findings decreased a misdiagnosis rate of RDS by 30.1% and subsequently resulted in a 30.1% reduction in PS use. (2) Among the 269 RDS patients diagnosed based on LUS findings, 148 were treated with Curosurf (another 121 RDS infants who received domestic PS treatment were not included in the study group), and the average dose was 105.4 ± 24.3 mg/kg per time, which is significantly lower than the dose of 200 mg/kg per time recommended by the European RDS guidelines. (3) The mortality rate of RDS patients was 0%, and no patients had ventilator-associated pneumonia or bronchopulmonary dysplasia in this study.

**Conclusion:**

LUS can decrease the misdiagnosis rate of RDS, thereby decreasing the probability of using PS and decreasing the dose of PS, and can help RDS infants to achieve better outcomes.

## Introduction

Respiratory distress syndrome (RDS) is a common and severe pulmonary disease in newborn infants caused by primary or secondary deficiencies in pulmonary surfactant (PS), which result in the formation of an eosinophilic hyaline membrane and atelectasis of the lungs from the alveolar wall to the terminal bronchiole wall. As a result, progressive dyspnea, expiratory moaning, cyanosis, and respiratory failure occur shortly after birth. The high incidence, rapid progression, and high mortality of RDS not only causes serious harm to premature infants but also damages the health of full-term infants (1–3). RDS not only significantly increases the incidence of pulmonary hemorrhage, pneumothorax, persistent pulmonary hypertension in newborns (PPHN), and other complications but further increases the fatality rate of RDS up to 43% (4, 5), while survivors also have an increased incidence of bronchopulmonary dysplasia (BPD) (5, 6). Therefore, exogenous PS supplementation is one of the main measures for the successful treatment of RDS (7–9), and the recommended dose of PS is 200 mg/kg per time (8). It was reported that the misdiagnosis rate of RDS was as high as 62%–77% according to traditional diagnostic criteria (10, 11). Recently, lung ultrasound (LUS) has been effectively applied in the diagnosis of RDS with more accuracy and reliability than chest x-ray (CXR) (12–14). This paper explored whether the application of PS under LUS monitoring can achieve the objectives of decreasing the misdiagnosis rate of RDS and subsequently decreasing the probability of PS using, as well as reducing the PS dose without reducing the therapeutic effect.

## Patients and methods

This study was approved by the Ethics Committee of Beijing Chaoyang District Maternal and Child Healthcare Hospital (No. 2011-LC-Ped-01). All methods were performed in accordance with the relevant guidelines and regulations. Written informed consent was obtained from the participants’ parents.

Since March 2017, patients admitted to the Department of Neonatology and NICU at Beijing Chaoyang District Maternal and Child Healthcare Hospital diagnosed with RDS and requiring PS treatment based on conventional criteria were included in this study.

This study included two parts:

*Part 1: To decide whether the LUS is good to differentiate RDS from other lung diseases in the premature infants*: All patients who met the above criteria underwent LUS examination to determine how many of them still needed PS treatment based on their LUS presentation (12–14). Thus, it can be determined whether LUS monitoring can decrease the misdiagnosis rate of RDS and decrease the probability of PS using.

*Part 2: To see the dose reduction of surfactant is applicable:* In RDS patients diagnosed based on LUS findings and treated with Curosurf (Chiesi Pharmaceutical, Parma, Italy), the Curosurf dose used in this study was compared with that recommended by the European RDS management guidelines (8), to determine whether LUS monitoring is helpful in reducing the dose of PS. At the same time, the outcomes of RDS patients, including mortality rate and complications such as ventilator-associated pneumonia (VAP) or BPD, were recorded.

**Exclusion criteria:** (1) Nonuse of surfactants: Patients who did not meet the ultrasound diagnostic criteria for RDS. (2) The PS agent used was domestic Calsurf (one kind of PS agent derived from calf lung tissue, made in China) but not Curosurf [because of (1) absence of internationally recognized therapeutic dose as a control and (2) lack of a strict case–control study].

**LUS examinations**: The GE Voluson S10 (GE Healthcare, United States) ultrasound system with a linear array probe (frequency >10 MHz) was used for LUS examinations in this study. All examinations were strictly performed in accordance with published guidelines and specifications (15). The criteria for LUS diagnosis of RDS were as follows (12, 13, 16, 17): (1) Lung consolidation with air bronchograms: early-stage or mild RDS can be characterized by the ground-glass opacity sign (GOS), and progressive or severe RDS is characterized by snowflake signs (SFS); (2) The pleural line is abnormal, and the A-lines disappear; (3) Double lung point; (4) Pleural effusion; and (5) Inconsistencies in the lesion degree and pathological properties of different parts of the lung.

**Statistical analysis:** The Statistical Package for the Social Sciences (SPSS) 24.0 software was used to statistically analyze the data. Count data are expressed as percentages (%), and measurement data are expressed as the mean ± standard deviation (mean ± SD). A value of *p* < 0.05 indicated statistically significant differences if needed.

## Results

### The effects of LUS on the misdiagnosis rate of RDS and the utilization rate of PS

**Figure 1:**
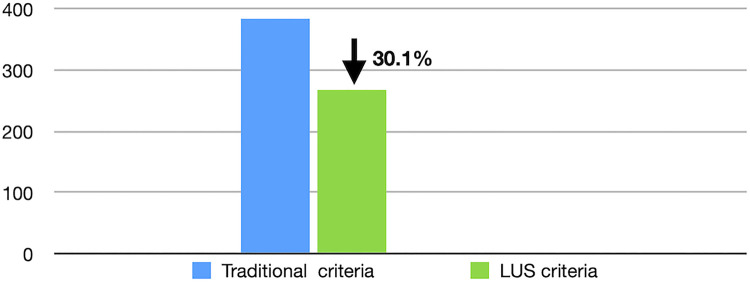
LUS decreases the misdiagnosis rate of RDS. Based on LUS diagnostic criteria, the misdiagnosis rate of RDS decreased 30.1% and correspondingly the application of exogenous PS decreased by the same proportion. LUS, lung ultrasound; RDS, respiratory distress syndrome; PS, pulmonary surfactant.

During the study period, 385 newborn infants met the traditional diagnostic criteria of RDS. Of these patients, only 269 infants met the ultrasound diagnostic criteria for RDS and required PS treatment, while the other 116 patients did not receive PS treatment but had good outcomes. This result means that LUS decreased the misdiagnosis rate of RDS by 30.1% and correspondingly decreased the application of exogenous PS by the same proportion (Figure 1). The additional 116 patients were diagnosed with transient tachypnea of the newborn (TTN) in 82 cases (16–20), pneumonia in 21 cases (16, 21), and meconium aspiration syndrome (MAS) in 13 cases (16, 22), respectively, based on their LUS characteristics.

### Typical case of LUS guiding the PS application

**Figure 2:**
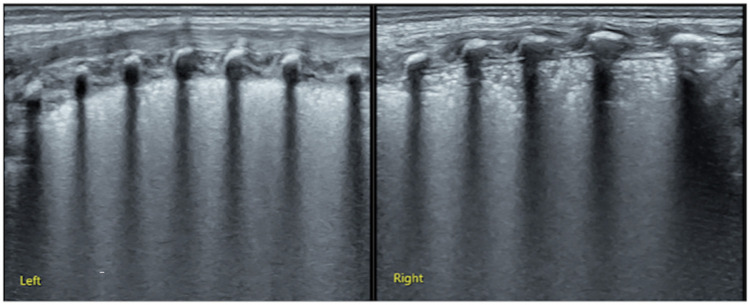
LUS examination results of Supplementary Video S1 (case 1). LUS showed snowflake-like lung consolidation in both lungs which is the typical ultrasound imaging of RDS, confirming that this patient needed PS treatment. LUS, lung ultrasound; RDS, respiratory distress syndrome; PS, pulmonary surfactant.

*Case 1*: This baby was G1P1 with a gestational age (GA) of 30 weeks, cesarean delivery, and birth weight (BW) of 1,800 g. The patient was admitted to our NICU because of progressive dyspnea and expiratory moans for 2 h. Significant retraction was noted on physical examination (Supplementary Video S1). Arterial blood analysis showed PaCO_2_: 57 mmHg, PaO_2_: 44 mmHg, and SaO_2_: 77%. Reduced transmissibility of both lungs and a bronchial air sign were noted on CXR in a local hospital. After admission, LUS examination showed SFS-like consolidation in the bilateral lungs, which was consistent with the ultrasound imaging characteristics of RDS (13, 16, 17, 23) (Figure 2). Therefore, this infant was treated with PS and recovered soon after.

**Figure 3:**
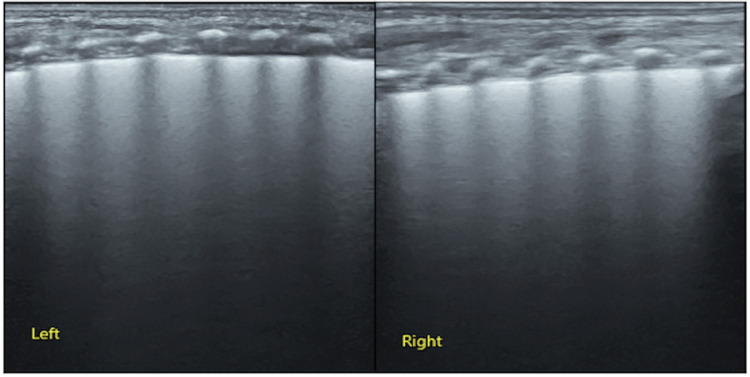
LUS examination results of Supplementary Video S2 (case 2). LUS showed confluent B-lines in both lungs suggesting significant lung edema, which is typical of ultrasound imaging of TTN. LUS, lung ultrasound; TTN, transient tachypnea of the newborn.

*Case 2*: This baby was G2P2 with a gestational age of 31 weeks, IVF, twin 2, and birth weight 1,620 g. The patient was admitted to our NICU because of progressive dyspnea and expiratory moans for 45 min. Significant retraction was noted at physical examination (Supplementary Video S2). Arterial blood analysis showed PaCO_2_: 55 mmHg, PaO_2_: 47 mmHg, and SaO_2_: 80%. Reduced transmissibility of both lungs and a bronchial air sign were noted on CXR. Therefore, the baby was also clinically diagnosed with RDS. However, LUS examination showed confluent B-lines in both lungs at admission, which was consistent with the ultrasound imaging characteristics of TTN but not RDS (17–19, 24) (Figure 3). Therefore, this infant did not receive PS treatment and recovered quickly.

### The initial dose of PS applied under LUS guidance

**Figure 4:**
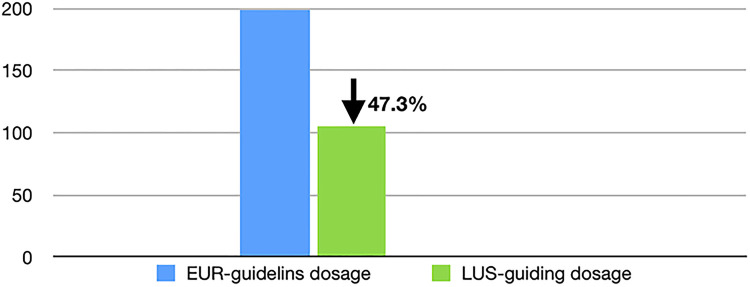
LUS monitoring decreases the initial dose of PS. As shown in Figure 4, when PS was applied under LUS monitoring, the initial dose used was decreased by nearly half compared with the dose recommended by the International Guidelines. LUS, lung ultrasound; PS, pulmonary surfactant.

The demographic data are shown in Table 1. Among the 269 RDS patients diagnosed based on LUS findings, 121 patients treated with domestic Calsurf were excluded from this study group. The remaining 148 cases treated with Curosurf were included in the study group, because it has the regular dose recommended by the International Guidelines (8), and the initial dose used by these 148 patients was compared with the dose recommended by the International Guidelines (8). The results showed that the average first dose was 105.4 ± 24.3 mg/kg per time in this group, while the recommended dose by the European RDS Guidelines was 200 mg/kg per time (8). That is, the initial PS dose under LUS monitoring was decreased by 47.3% compared to the International Guideline's recommended dose (Figure 4).

**Table 1:** The demographic data in different groups.

Groups	N	Gender (male/female)	Premature infants			Term infants		
			N	GA (range, weeks)	BW (range, g)	N	GA (range, weeks)	BW (range, g)
Calsurf	121	127/47	89	32.1±2.1 (27+2–36+6)	1,670.6±487.1 (850–2870)	32	38.0±1.2 (37–41+2)	2,538.1±943.0 (1100–4150)
Curosurf	148	15/16	126	32.6±2.6 (26+6–36+5)	1,814.8±614.6 (800–3170)	22	38.1±1.2 (37–41)	2,617.3±848.9 (1750–3710)
Total	269	147/111	215	32.5±2.5 (26+6–36+6)	1,755.1±569.1 (800–3170)	54	38.0±1.2 (37–41+2)	2,570.3±898.4 (1100–4150)

GA, gestational age; BW, birth weight.

### Effect of LUS monitoring on repeat doses of exogenous PS in RDS patients

**Figure 5:**
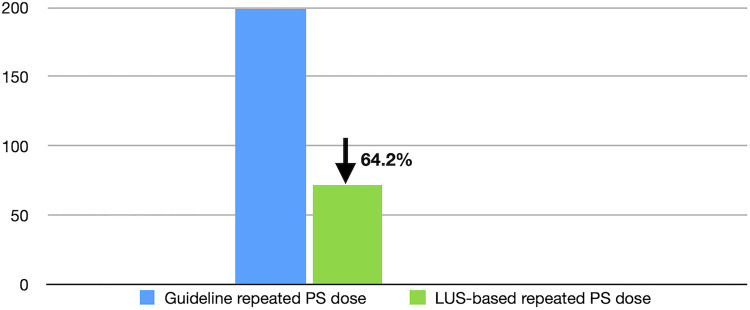
LUS monitoring decreases the repeat dose of PS. As shown in Figure 5, when PS was applied under LUS monitoring, the repeat dose was decreased by 64.2% compared with the dose recommended by the International Guidelines. LUS, lung ultrasound; PS, pulmonary surfactant.

Of the 148 RDS patients who received Curosurf treatment, 24 infants received repeat doses of PS supplementation. The repeated application dose was 71.6 ± 31.5 mg/kg per time. Therefore, the repeat dose was 64.2% lower than that recommended by the International Guidelines (8) (Figure 5).

### Patient outcomes based on LUS guidance management

No patient died, and no VAP or BPD complications occurred among these patients, which resulted in a mortality rate of 0% and a VAP and BPD incidence rate of 0%.

## Discussion

As mentioned above, the misdiagnosis rate of RDS based on the so-called traditional “Gold Standard” diagnosis can be as high as 62%–77% (10, 11). According to the results of this study, among the 385 infants diagnosed with RDS by traditional criteria, 116 were not diagnosed with RDS but with TTN, MAS, or pneumonia by LUS examination. Importantly, although these infants did not receive PS treatment, and they all had a good prognosis and outcomes. The results of the present study confirmed that the LUS is good to differentiate RDS from other lung diseases in the newborn infants, that is, LUS is more accurate and reliable than CXR in diagnosing lung diseases (25); the results of this study means that the routine application of LUS decreases the misdiagnosis rate of RDS by 30.1%. Subsequently, LUS helped us to decrease PS use rate by 30.1% in the present study. PS, as we all know, is a very expensive agent. The reduction of its use not only decreases patients’ medical costs but also saves medical resources.

PS is an effective agent for the treatment of RDS, but the dose of PS has been controversial. The recommended dose of PS is 200 mg/kg per time according to the European guidelines (8). The results of this study showed that the PS dose used in RDS patients was 105.4 mg/kg per time on average; this dose could be half (47.3%) of the guideline recommended dose (8). Another important finding was that the repeat dose was reduced by 64.2% compared with the guideline recommended dose (8). Most importantly, all of the patients achieved a good outcome without any death or complications, such as MAS or BPD, when using the lower dose. Therefore, the use of LUS monitoring to guide PS application can not only decrease the misdiagnosis rate of RDS and decrease the usage rate of PS but also decrease the initial and repeat dose needed, which together further demonstrates the socioeconomic value of performing lung ultrasound (25).

It has been reported that the LUS score should be used to determine whether a newborn infant with dyspnea should receive PS treatment (26, 27). There is no doubt that LUS scoring has some value in some cases, but it is complex, time-consuming, and is subject to a variety of factors, including operators’ subjective factors and the instrument itself (28). According to the ultrasound performance of the infants, we can easily and directly determine whether a patient with dyspnea needs to receive PS treatment. This method is simpler, more intuitive, and easier to apply in critical and severe patients. That is, if LUS shows GOS-like or SFS-like lung consolidation, PS can be directly used for patients, which are the specific ultrasound signs of RDS (17, 22). Reexamining the LUS 2–4 h after initial PS treatment, if the lung lesion does not alleviate or even becomes more severe, PS should be repeated for the patients.

There are some limitations in this study. The GA and BW of the infants are relatively large. The proportion of infants with GA <27 weeks and BW <1,000 g accounted for only a small proportion, which may reduce the chance of poor prognosis; further research needs to focus more on babies at lower gestational ages and birth weights. If one wants to perform LUS well, he or she needs to receive systematic training. In addition to mastering the basic theory of LUS, he or she needs to master operating skills and instrument adjustment skills (15). In addition, a high-frequency linear array transducer should be used to make an accurate diagnosis (15).

In summary, the result of the present study showed that LUS can help us to decrease the misdiagnosis rate of RDS, thereby decreasing usage and dose of PS, and can help RDS infants achieve better outcomes. Thus, it deserves widespread application in clinical practice.

## Data Availability

The original contributions presented in the study are included in the article/Supplementary Material, further inquiries can be directed to the corresponding author.

## Supplementary material

Supplementary Video S1 Case 1: A premature infant with respiratory dyspnea. This was a premature infant with a gestational age of 30 weeks and birth weight of 1,800 g who was admitted to the NICU because of progressive dyspnea, expiratory moans, and significant retraction. The blood gas analysis was significantly abnormal.

Supplementary Video S2 Case 2: A premature infant with respiratory distress. This was a baby with a gestational age of 31 weeks and birth weight of 1,620 g who was admitted to the NICU because of progressive dyspnea, expiratory moans, and significant retraction. The blood gas analysis was significantly abnormal.
